# Prenatal Alcohol Exposure and Neurobehavioral Development: Where Is the Threshold?

**Published:** 1994

**Authors:** Joseph L. Jacobson, Sandra W. Jacobson

**Affiliations:** Joseph L. Jacobson, Ph.D., is professor of psychology in the psychology department, Wayne State University, Detroit, Michigan. Sandra W. Jacobson, Ph.D., is research professor of psychology in the psychology department, Wayne State University, Detroit, Michigan

## Abstract

In terms of prenatal alcohol-induced alterations in neurobehavioral outcomes such as attention, activity level, and information-processing speed, a threshold of alcohol consumption may be difficult to determine. Studies on humans indicate that seven standard drinks per week may be the threshold for the most sensitive neurobehaviors but may not apply to all women and all babies.

Much of the research on prenatal alcohol exposure has focused on the hypothesis that drinking during pregnancy causes structural and biochemical alterations in the developing brain of the fetus. These alterations are thought to affect many aspects of intellectual and behavioral function, including attention, activity level, and information-processing speed. Deficits in these aspects of neurobehavioral functions can be measured by using a variety of assessment instruments on children at various ages from infancy to late childhood.

One important issue is the level of maternal drinking necessary before neurobehavioral impairment is seen. Because the body has the ability to tolerate low doses of most toxic substances, adverse effects are seen only when exposure exceeds a certain minimum threshold dose. Severe effects of alcohol, such as fetal death, mental retardation, and the craniofacial deformities associated with fetal alcohol syndrome (FAS), have relatively high thresholds, occurring only in the most heavily alcohol-exposed fetuses. Sokol and colleagues (1988) estimate that the threshold for FAS is consumption by the mother of approximately 42 standard drinks (21 ounces of absolute alcohol) per week around the time of conception (table 1). Even at this level, however, not all children will exhibit FAS (Sokol et al. 1986).

Only a few studies that have focused on the effects of alcohol on neurobehavioral development have investigated thresholds (Jacobson et al. 1993; Streissguth et al. 1983). Most of the initial data on thresholds for neurobehavioral effects came from a Seattle cohort of children studied by Streissguth and colleagues (1983).

These children were born to approximately 500^1^ predominantly white, middle-class women in the mid-1970’s. In contrast, a recent study conducted in Detroit (Jacobson et al. 1993) examined 480 economically disadvantaged, black infants. In both studies, mothers were recruited during pregnancy. All moderate to heavy drinkers and randomly selected lower level drinkers and abstainers were invited to participate (see table 1 for drinking levels).

The data from these two longitudinal^2^ cohort studies form the basis of this article. We describe three types of dose-response relationships reported for drinking during pregnancy, introduce alternative approaches for determining threshold values, review data on the thresholds found for neurobehavioral outcomes, and consider the policy implications of estimating thresholds.

## Dose-Response Patterns

**Figure 1:**
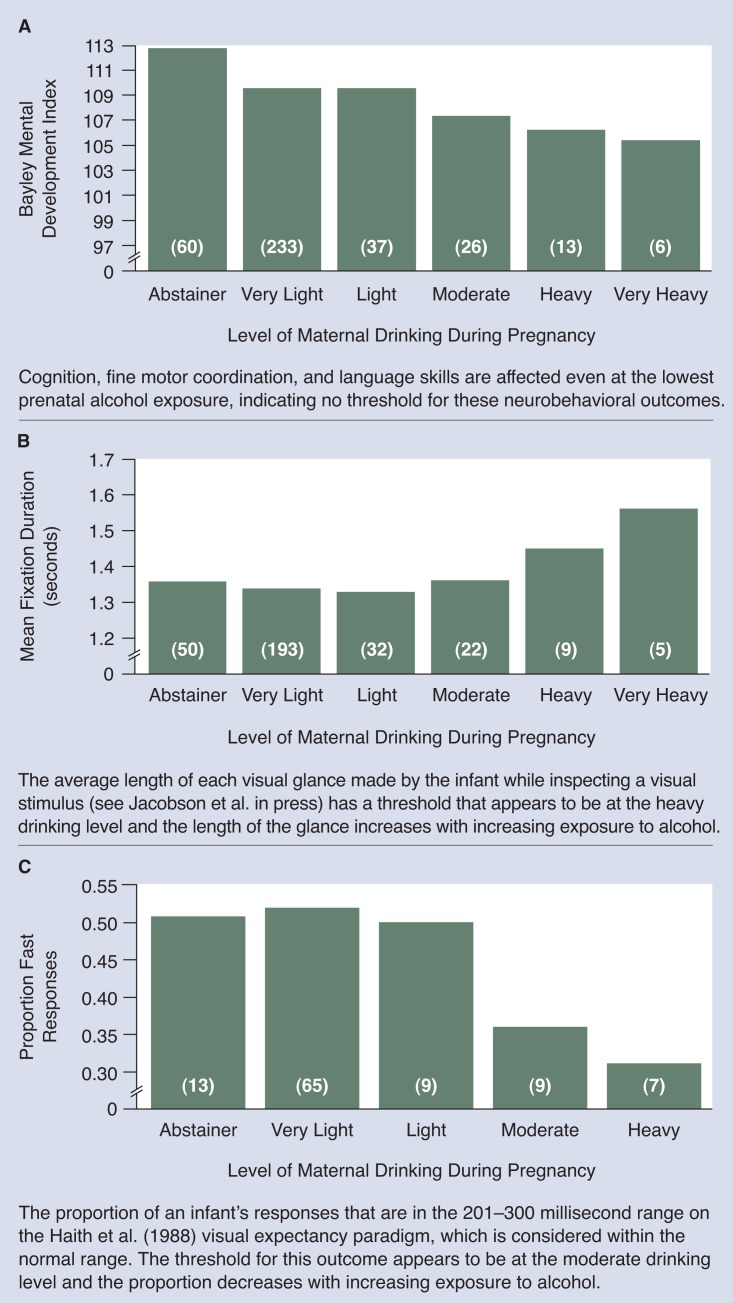
Dose-response effect of maternal drinking during pregnancy on three infant neurobehavioral outcomes. The number of children in each drinking level exposure group is given in parentheses (see table 1 for actual amounts of alcohol).

For some behaviors, such as mental development, even the smallest dose of alcohol prenatally appears to have some adverse effect on the fetus, and the severity of the effect increases gradually with increasing levels of exposure (see figure 1A). This dose-response relationship can be described as “linear with no apparent threshold.” However, most neurobehavioral outcomes are not so sensitive. Rather, they seem to have relatively high thresholds, above which the effect then becomes more severe with increased exposure (figures 1B and 1C).

In some studies, step functions have been reported. As in figures 1B and 1C, little effect is seen below an observed threshold, but, unlike figures 1B and 1C, the effect does not appear to become more severe with increased exposure above the threshold. The effect occurs in one “step” at the threshold level of alcohol consumption and remains steady, regardless of how high consumption rises above that level. Although the step-function pattern is fairly common in alcohol studies, it does not make complete sense toxicologically, because severity of effect should increase with additional exposure. In some cases, the appearance of a step function may be a result of not including enough heavily exposed children in the sample, which can lead to an underestimate of the severity of the effect at the highest doses.

## Determination of Dose-Response and Threshold

Thresholds can be determined more precisely with laboratory animals than with humans. In animal studies, the amount of prenatal alcohol exposure can be carefully monitored and controlled, and potentially confounding factors, such as poor diet and exposure to other harmful substances (e.g., smoking), can be prevented. In humans, information on prenatal alcohol consumption is obtained by self-reports, and confounding factors cannot be eliminated. In other words, if a neurobehavioral deficit is found in a child, statistical analyses must be used to determine whether prenatal alcohol consumption is responsible for the deficit instead of smoking, poor eating habits, or poor parenting post partum.

### Animals

**Figure 2:**
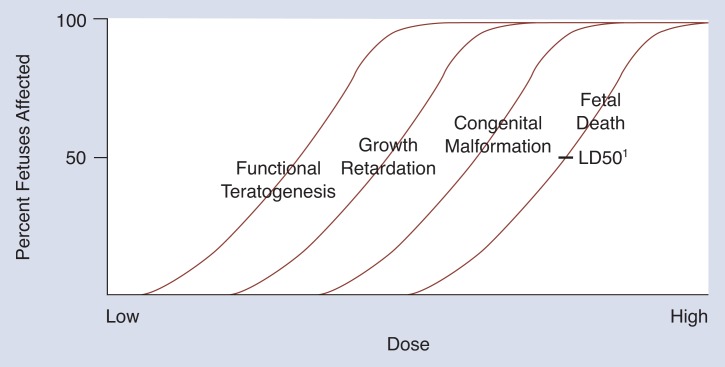
Ideal dose-response curves for four domains affected by toxic exposure during fetal development. As dose of a toxic substance increases, more fetuses are at risk of injury and effects become more severe, ranging from functional teratogenesis, which includes neurobehavioral outcomes, to fetal death. ^1^The LD50 represents the median lethal dose of a toxic substance at which half of the fetuses exposed will die. SOURCE: Vorhees 1986.

Animal studies are used to determine a median lethal dose (LD50) of a toxic substance—the dose at which half of the fetuses exposed will die. It is assumed that individual differences in sensitivity to the exposure are distributed around the LD50; some fetuses will die at a lower dose and others will survive an even higher dose (Klaassen 1986). If a large number of different doses are tested, with a large number of animals per dose, an S-shaped curve is observed (represented by the fetal death curve in figure 2). The threshold is the dose of alcohol at which all but the most vulnerable fetuses are affected. Certain outcomes are expected to have lower thresholds than others. Death would be expected to take place at the highest threshold, whereas milder forms of damage would occur at lower doses (see figure 2).

### Humans

#### Data Collection

Two longitudinal studies have found that levels of pregnancy drinking reported by women who were interviewed after giving birth were markedly higher than those reported by women who were interviewed during pregnancy (Ernhart et al. 1989; Jacobson et al. 1991). Ernhart and colleagues (1989) found that one developmental outcome—physical anomalies, such as facial and limb defects—was predicted better by reports of pregnancy drinking taken 5 years after giving birth than by drinking reports obtained during pregnancy. The data from Jacobson and colleagues’ Detroit study, on the other hand, suggest that drinking reports obtained during pregnancy are more accurate. Retrospective reports obtained at 13 months post partum were only weakly related to birth size and did not relate to any of the neurobehavioral outcomes shown in table 2, all of which were associated with reports of drinking during pregnancy (e.g., Jacobson et al. 1993). The thresholds reviewed here are based on maternal drinking reported during pregnancy.

#### Data Analysis

In the Detroit study, we used the statistical technique of multiple regression analysis to test whether prenatal alcohol exposure adversely affects the neurobehavioral outcomes assessed. This technique is used to rule out other potentially harmful factors, such as smoking, that may occur along with pregnancy drinking. A broad range of such potential confounding influences, including amount of prenatal care, smoking and illicit drug use during pregnancy, and quality of parenting, were measured and, where necessary, controlled for with the regression analysis (see Jacobson et al. 1993). A neurobehavioral deficit was attributed to prenatal alcohol exposure only when the odds were less than 1 in 20 (*p* < 0.05) that the deficit was due to chance after adjustment for the effects of the potential confounders. If a neurobehavioral outcome was found to be influenced significantly by alcohol exposure, it was subsequently evaluated for a threshold to assess the level of alcohol exposure at which a response was seen.

To evaluate dose-response patterns, we separated levels of alcohol exposure into discrete groups and plotted group means for each neurobehavioral outcome at each exposure group (adjusted statistically for potential confounding influences) (figure 1). To create exposure groups, we started with the consumption levels used in the 1988 National Health Interview Survey: abstainer, light, moderate, and heavy (for actual quantities of alcohol consumed at these levels, see table 1). Given the large number of subjects available in the light group, we subdivided that group by creating a “very light” group. Also, for some developmental outcomes, data were available for a sufficiently large number of heavily exposed infants to create a “very heavy” group. Because virtually all of the moderate- and heavy-drinking women concentrated their drinking on a few days each week, the values used for plotting are presented in terms of ounces of absolute alcohol per week.

In the Seattle study, mothers were interviewed during the fifth month of gestation about their drinking during the “month or so prior to pregnancy recognition” and during pregnancy. The association between neurobehavioral deficits and early pregnancy drinking was generally stronger than the association between neurobehavioral deficits and midpregnancy drinking (e.g., Streissguth et al. 1984), leading the researchers to suggest that the human infant might be particularly sensitive to alcohol exposure during early pregnancy. However, in the study of Detroit infants exposed to alcohol at similar levels, neurobehavioral effects were associated more strongly with later pregnancy drinking than with drinking around the time of conception.

The Detroit study used a more detailed self-report procedure, in which mothers were interviewed at each prenatal clinic visit regarding their drinking on a day-by-day basis during the preceding 2 weeks. The interviewer started out by asking the following questions:

I would like you to think back to last Friday. What did you do? Did you go out? Did you relax in front of the TV? Did you have a drink? What were you drinking that evening? How do you drink that beverage? By the can? By the glass? How big a glass do you usually drink?

The stronger relationships between pregnancy drinking and neurobehavioral deficits in the Detroit study suggest that the detailed interview procedure may provide a more reliable assessment of pregnancy drinking than the one-time midpregnancy report used in Seattle.

## Review of Findings

The neurobehavioral outcomes tested for thresholds in the Seattle study are summarized in table 3. Although some measures were found to have no threshold, notably for reaction times at 4 and 7 years of age, most measures appear to have thresholds ranging from 7 to 28 standard drinks per week, as measured prior to pregnancy recognition or at midpregnancy. The thresholds detected for neurobehavioral outcomes in the Detroit study (table 2) are in the same range, although the values on certain measures, such as mental and motor development, differ between the two studies. The data from both studies suggest that most adverse neurobehavioral effects are not seen below seven standard drinks per week. In fact, studies that have included mostly mothers who drink fewer than seven standard drinks per week during pregnancy generally have failed to detect effects on neurobehavioral development in infancy (e.g., Greene et al. 1991), supporting the suggestion that seven standard drinks per week is a threshold level for most neurobehavioral effects (Jacobson et al. 1993).

Although the neurobehavioral outcomes shown in tables 2 and 3 were tested statistically to ensure that the alcohol effect is not attributable to other confounding factors, the specific threshold values listed in these tables were not all tested for significance. In many cases, the number of children exposed to alcohol above the threshold was too small to test for statistical significance. The conclusion that the threshold for neurobehavioral effects lies in the range of 7 to 28 standard drinks per week is based on the consistency of the data across a large number of neurobehavioral outcomes rather than on the statistical significance of the individual threshold values observed.

It should be noted that because few pregnant women drink every day, seven standard drinks per week typically represents relatively heavy doses of alcohol on drinking days. For example, the mothers in the Detroit sample who drank more than 7 standard drinks per week exposed their infants to an average of 6 drinks per day (at a range of 1.2 to 24.8 drinks) on an average of 2.6 days per week (at a range of 0.6 to 7.0 days) (Jacobson et al. 1993).

Alcohol’s adverse effects on the children’s neurobehavior were by no means limited to the children of alcohol abusers. The majority of the Detroit mothers who drank more than seven standard drinks per week were negative on the Michigan Alcoholism Screening Test (MAST), indicating that their drinking was not marked by the psychosocial sequelae of alcohol abuse. Even those moderate- and heavy-drinking mothers whose infants performed poorly on the tests shown in table 2 (i.e., in the bottom 10th percentile of the distribution) were no more likely to be positive for alcoholism on the MAST than those mothers whose infants performed adequately or well.

## Neurobehavioral Outcome Measures

According to Vorhees (1986) and others, neurobehavioral outcomes appear to be the most sensitive index of the fetal toxicity, or teratogenicity, of a toxic substance that affects multiple developmental domains (e.g., fetal death, congenital malformations, growth retardation, and neurobehavioral function) (figure 2). Levin and colleagues (1992) have pointed out, however, that structural changes in the brain can now often be detected at even lower levels of exposure than are neurobehavioral deficits. For example, in animal studies of halothane (a surgical anesthetic) exposure during development, structural abnormalities in the connections between the nerve cells of the hippocampus were evident at much lower levels of exposure than the short-term memory deficits traditionally associated with damage to that brain area. Such findings indicate that extremely low levels of teratogens, such as halothane and alcohol, may cause structural damage not severe enough to cause obvious effects on function. Improvements in the ability to measure neurostructural changes more precisely make it likely that such changes will be seen at increasingly lower doses. Ultimately, however, the information of greatest interest is the lowest dose at which effects are seen that have a meaningful impact on neurobehavioral or other *function*.

Within the broad domain of neurobehavior, specific areas of function (e.g., motor coordination, activity level, sustained attention) are likely to vary in their sensitivity to different toxic agents. For example, in the Detroit study, the threshold for the 13-month Bayley Psychomotor Development Index, which assesses gross motor development, was much higher than the threshold for the Bayley Mental Development Index, which assesses cognition, fine motor coordination, and language skills (table 2).

Thresholds vary not only by functional domain but also within domains, depending on the sensitivity of the measures used. For example, two measures of cognitive-processing speed were used in the Detroit study. The first, mean duration of visual fixation, measures the average length of time that an infant looks at each of two pictures or objects placed side by side. Infant development researchers have found that in infancy, shorter looks are associated with more rapid encoding of visual information (Colombo et al. 1991) and predict higher childhood IQ scores (Sigman et al. 1991). Thus, shorter looks apparently indicate the ability to assimilate and process information more quickly.

The second measure of infant cognitive-processing speed used in the Detroit study was reaction time in shifting gaze back and forth in response to an image flashing in alternating left-right positions on a video screen (Haith et al. 1988). Reaction time also appears to reflect speed of information processing and, in fact, infants with slower times on the fixation-duration measure also respond more slowly to the onset of the visual stimulus in the reaction-time measure (Jacobson et al. 1992*a*).

Prenatal alcohol exposure was associated with slower processing speed on both speed of processing measures (Jacobson et al. in press; Jacobson et al. 1992*b*); however, the reaction-time measure proved markedly more sensitive, detecting effects at 7 drinks per week (figure 1C), compared with a 14-drinks per week threshold for fixation duration (figure 1B). Similarly, the threshold for effects on IQ at 7 years (Streissguth et al. 1989) was lower than at 4 years (Streissguth et al. 1990), presumably due to the superior reliability of the test at the older age (table 3). These data show the extent to which neurobehavioral threshold values depend on the sensitivity and reliability of the testing instruments.

## Functional Significance

As noted earlier, some neurobehavioral measures appear to have no threshold; they are so sensitive that effects are seen even at extremely low levels of alcohol exposure. For example, the data in figure 1A suggest that the mental development of infants whose mothers drank at light levels (an average of 0.85 standard drinks per week during pregnancy) is poorer than that of the infants of abstainers. However, because reductions in neurobehavioral scores may be only a few points or fractions of a second at low doses, particular consideration should be given to the functional significance of alcohol’s neurobehavioral effect. That is, to what extent does the deficit have a meaningful impact on the child’s ability to acquire information or to perform an intellectual or motor task?

Criteria for functional significance exist for some standardized measures, such as IQ, but not for experimental measures, such as the fixation-duration and reaction-time measures described above. Even on standardized tests, established criteria identify mental retardation or “borderline” retardation but cannot be used to evaluate the functional significance of lower scores within the normal range. In the absence of established criteria, we use the bottom 10th percentile of the distribution or one standard deviation below the mean as provisional criteria for determining the degree to which exposure to alcohol is associated with an increased incidence in what might be considered poor performance on a test.

Table 4 shows the incidence of poor mental development performance (i.e., number of low scores) at each level of prenatal alcohol exposure. In contrast with the analysis that compares group means (figure 1A), which suggested subtle effects at even the lowest levels of exposure, the analysis based on the poor performance criterion indicates no increased incidence in functional impairment below seven standard drinks per week. Below this threshold, the rate of poor performance was in the range of what might be expected by chance.

Researchers are generally reluctant to set criteria for functional significance. To evaluate properly the functional significance of scores on infant tests, detailed prospective longitudinal data are needed to determine what level of infant performance is associated with poor performance later in childhood. Even if such data were available, the limited predictive power of most infant measures would raise questions about the usefulness of the criteria selected. In childhood, the degree to which performance on a test is indicative of functional limitations outside the testing situation remains a matter of clinical judgment, which quantitatively oriented researchers are usually hesitant to make.

## Implications for Defining Threshold

In human neurobehavioral studies, threshold is usually defined in terms of the level of exposure to a toxin below which average *group* performance is not adversely affected. As illustrated by data from laboratory experiments with animals, however, there are typically marked individual differences in vulnerability to any given exposure (figure 2). Because the threshold values derived from human studies are based on group averages, it is not appropriate to infer that exposure just below a threshold level is necessarily “safe,” because some individuals could be markedly more sensitive than others.

In evaluating risk associated with exposure to environmental and food contaminants, a margin of safety is usually incorporated to allow for individual differences in sensitivity. Where human data are available, a margin of safety of a factor of 10 is used for this purpose (Sette and Levine 1986). Taking this approach, it is possible to divide the threshold value of 3.5 ounces of absolute alcohol per week by 10 and conclude that 0.35 ounces per week (one drink every 10 days) during pregnancy is likely to be “safe.” On the other hand, even if no functional deficits are associated with a given level of exposure in infancy and childhood, there is the potential for unobservable neurostructural damage, which could lead to functional deficits when the child is stressed or challenged by a complex task (Riley 1990), or when the child reaches old age.

Because alcohol exposure has no apparent benefit for the developing fetus and is not necessary for the health and well-being of the mother, some clinicians and health officials have argued that the best policy is to advise pregnant women not to drink at all during pregnancy.

Given the relatively high levels of alcohol at which any functionally significant deficits have been documented, however, other clinicians find it difficult to justify the need for complete abstinence. The Surgeon General, however, has advised that all pregnant women abstain from drinking throughout pregnancy, because there is no way to determine definitively which babies may be at risk for damage from very low levels of alcohol exposure.

## Figures and Tables

**Table 1:** Drinking Levels in Ounces of Absolute Alcohol per Week and Standard Drinks^1^ per Week

Drinking Level	Ounces Absolute Alcohol/Week	Standard Drinks/Week
Abstainer	0	0
Very Light^2^	0.01–1.74	0.02–3.49
Light^2^	1.75–3.49	3.50–6.9
Moderate	3.50–6.99	7.00–13.9
Heavy^3^	7.00–13.99	14.00–27.9
Very Heavy^3^	14.0 +	28.0 +

1 One standard drink ≅ 0.5 oz absolute alcohol ≅ 12 oz beer ≅ 5 oz wine ≅ 1.25 oz liquor.

2 These categories compose the “light” category in the National Institutes of Health (NIH) 1988 National Health Interview Survey.

3 These categories compose the “heavy” category in the NIH 1988 National Health Interview Survey.

**Table 2:** Thresholds at Which Neurobehavioral Deficits Were Seen for the Detroit Cohort

Standard Drinks per Week	Neurobehavioral Outcome	Age of Children at Measurement
28	Delayed gross motor development	13 months^1^
	More prolonged episodes in play with a toy	12 months*
14	Slower information-processing speed	6.5 and 12 months*
7	Delayed mental development	13 months^1^,^2^
	Slower reaction time	6.5 months^3^
	Smaller proportion of fast responses	6.5 months^3^
No threshold (Fewer than 7 drinks)	Less complex play	12 months*

1 Jacobson et al. 1993.

2 Threshold appears only when this outcome is examined in terms of the proportion of infants who exhibit poor performance (see table 4).

3 Jacobson et al. 1992*b*.

* Jacobson et al. in press.

**Table 3:** Thresholds at Which Neurobehavioral Deficits Were Seen for the Seattle Cohort

Standard Drinks per Week	Neurobehavioral Outcome	Age of Children at Measurement
Prior to Pregnancy Recognition		
28	Delayed mental development	8 months^1^
	Delayed gross motor development	8 months^1^
	Greater impulsivity	7 years*
21	Lower IQ scores	4 years^2^
	Poorer fine motor coordination	4 years†
	Poorer sustained attention	7 years*
14	Poorer sustained attention	4 years‡
7	Poorer fine motor coordination	4 years†

Midpregnancy		
28	Poorer habituation^3^	1–2 days^4^
21	Poorer fine motor coordination	4 years†
14	Lower IQ scores	7 years^5^
No threshold (Fewer than 7 drinks)	Slower reaction times	4 years†,‡
	More impulsive behavior	4 years‡
	Poorer balance in standing and walking	4 years†
	Slower reaction time	7 years*

1 Streissguth et al. 1980.

2 Streissguth et al. 1989.

3 Habituation, an important component of attention, is the ability to stop attending to a repeatedly presented stimulus.

4 Streissguth et al. 1983.

5 Streissguth et al. 1990.

* Streissguth et al. 1986.

† Barr et al. 1990.

‡ Streissguth et al. 1984.

**Table 4:** Infants by Pregnancy Drinking Level Scoring in the Bottom 10th Percentile on a Mental Development Index

Pregnancy Drinking Level^1^	Total Infants in Each Drinking Level Exposure Group	Number of Infants in Each Drinking Level Exposure Group Scoring in the Bottom 10th Percentile	Percent of Total Infants in Each Drinking Level Exposure Group Scoring in the Bottom 10th Percentile
Abstainer	60	4	6.7
Very Light	233	22	9.4
Light	37	2	5.4
Moderate	26	6	23.1
Heavy	13	2	15.4
Very Heavy	6	1	16.7
Total	375	37	9.9

1 See table 1 for actual amounts of alcohol.

SOURCE: Jacobson et al. 1993

## Footnotes

1 The number of women fluctuated at different phases of the study.

2 Longitudinal studies examine the same group of people at intervals over time as they age.
